# Some Varieties and Their Treatment

**Published:** 1919-11-15

**Authors:** 


### CHRONIC DIARRHŒA.

### Some Varieties and their Treatment.

This was the subject of the lecture delivered at the Royal Society of Medicine on September 23 by Dr. Robert Hutchison, of the London Hospital and the Hospital for Sick Children, in connection with the post-graduation course of the Fellowship of Medicine. In the following abstract we give the main points put forward:

The ordinary conception of diarrhœa is that it consists of an increase in the number of motions which are normal to the patient, but this is incomplete, because it really consists of a definite increase in fæcal matter passed, which may well happen even though there be but one motion *per diem*. To this latter kind the term "masked diarrhœa" is applied, and in this form, apparently, the food is unduly hurried through the small intestine, but the colon has no share in the hustle, hence there is an accumulation in the colon, and the action of the bowels is consequently a very large one.

The first kind of diarrhœa in which there is an increased number of motions is termed "spurious": it might almost be regarded as obstructive, seeing that it is produced by partial obstruction. One of its varieties is that in which there is obstruction by scybala, met with particularly in old people in whom the colon lacks tone. To treat such a condition by astringents would be the worst thing possible. Examination of the rectum—which should never be neglected in cases of diarrhœa—will reveal scybalous masses with much mucus. The treatment is to empty the bowel by means of enemata and the administration of castor oil. The other and far more important form of spurious diarrhœa is that due to malignant disease in the bowel, especially in the upper part of the rectum. These cases are often overlooked, because there is nothing in the patient's history or appearance to cause one to suspect the disease.

### The Medical Varieties.

Considering the medical forms of diarrhœa, according to origin, the first is gastrogenic, a type which is not as often recognised as it should be. The stomach faults are (1) a deficiency in its secretion, possibly even an absence of it; (2) a too rapid emptying of that viscus. This achylia may not be attended by any gastric symptoms; indeed, the patient may deny there is anything wrong with the stomach at all, and the only certain way of diagnosing it is by a test meal, though it can be ascertained whether the stomach empties itself too quickly by making an *x*-ray examination. There is nothing in the character of the motions to give a clue, except that, microscopically, they contain a fairly large proportion of undigested muscle fibre. If a test meal is objected to, give hydrochloric acid, and see what happens.

Next come the diarrhœas whose seat is the small intestine, *i.e.*, the enteritises, or catarrhs of the small intestine. These are, in this country, probably not a common cause of chronic diarrhœa in adults, though they may cause acute diarrhœa. If there are particles of mucus stained with bile, catarrh of small intestine is indicated. It rarely occurs as a primary disease; when it takes place it is usually secondary to disease elsewhere. For instance, it is seen in people with a cirrhotic liver, and in subjects of kidney disease. Primary catarrh of the intestine is not easy to treat, and no kind of case requires more individualistic treatment. Here, as in most cases of diarrhœa, diet will play a large part, and it is important to eliminate diet which produces irritation, either mechanical, chemical, or thermal irritation. The best drugs for this are the salts of calcium, and preparations of tannic acid are also useful in this form of diarrhœa.

The next variety is fermentative diarrhœa—*i.e.*, that due to the fermentation of carbo-hydrates, particularly starch. It is due to a deficiency of those ferments whose business it is to convert and digest starch, and so the starch in the food undergoes abnormal fermentation, acids are produced, and the patient passes sour, frothy, irritative stools. The treatment consists in cutting down the starch in the diet, or seeing that it is first partially converted into diastase, etc., also giving alkalis to neutralise the acidity.

The next form is the pancreatic, the diarrhœa here being due to deficient pancreatic secretion. One of the main functions of the pancreas being to digest fats, the stools, in this variety of diarrhœa, contain quantities of undigested fat, perhaps also of starch. The character of the motions will suffice to put the doctor on the scent. The treatment here is to reduce the fat in the dietary and give some pancreatic preparation.

### An Important Group.

We pass to the important group of cases which have their seat in the colon. The first of these true diarrhœas are those due to catarrhal colitis. These probably constitute the majority of cases of chronic diarrhœa. If the patient has resided in the Tropics, the cause may be dysentery. This is usually revealed by the tendency for the motions to occur chiefly in the mornings, hence the term "morning diarrhœa." In all forms of colonic diarrhœa the motions, at some time, contain mucus which is visible to the naked eye, and have nothing characteristic about them, but there may, however, be some slight thickening and tenderness over the sigmoid.

Chronic catarrhal colitis is by no means easy to treat, especially if, as is often the case, the condition has lasted some years. When the history is only of a few weeks' duration, it is well worth while to carry out a thorough course of lavage of the colon, with either normal saline or normal saline to which

**Chronic Diarrhœa**—(*continued*).

is added some 1 in 5,000 permanganate of potash. If the sigmoidoscope shows the sigmoid and pelvic colon to be involved, add ½ per cent. to 1 per cent of argyrol, a silver preparation. In old-standing cases the lavage is probably not worth the inconvenience to the patient; it is, then, rather a question of care in the diet, and avoiding chill, which will often bring on a relapse. It is not advisable, in these long-standing cases, to get rid of the diarrhœa altogether, because it has been for so long a sort of safety-valve to the patient. One can keep it in check by the judicious use of drugs, such as full doses of salicylate or carbonate of bismuth in the morning, with or without aromatic chalk, and, possibly, a few grains of Dover's powder. Only a few, because you must not tie the bowel up unduly. People who have had chronic catarrh of the colon for many years will complain that they have " lost confidence " in their bowels, hence they do not feel safe about taking a railway or other long journey. The best advice to such is that they should lock up the bowels for four or five hours by taking, for such occasions only, a dose of chlorodyne, or, if preferred, opium. There is here an associated psychic element.

The other great form of colon disease which causes diarrhœa is ulcerative colitis—and again I am not speaking of dysentery. The true pathology of non-dysenteric ulcerative colitis does not seem to be known, and it does not appear to be due to a specific microbe. It is more common in women than in men, and it occurs in comparatively early life—from twenty to forty years of age. It is characterised by an insidiously beginning diarrhœa, which increases, and finally leads to the passage of blood and mucus. And it tends to appear in bouts, clearing up in the interval. Each bout tends to be more severe than its predecessor, and it may be a highly dangerous condition, attended by fever and prostration. There is a more acute form, which leads quickly to the passage of large quantities of blood, rendering the patient anæmic in a short time. If you are in doubt, the sigmoidoscope will show you the bowel covered with small ulcers, sometimes so plentiful as to leave only narrow isthmuses of healthy mucous membrane between. The condition must be regarded seriously: sometimes death results. If the ulceration goes deep enough, there may be perforation, followed by peritonitis.

The treatment of this should be surgical—cæcostomy or appendicostomy, so that the whole length of the colon can be washed out daily. The ulcers cannot be got at in any other way. Where only the lower part of the bowel is involved, you can probably do much good by using a weak permanganate solution *per rectum*. An appendicostomy wound can easily be kept open: a patient has been known to irrigate his colon through such a fistula for five years without difficulty.

